# BioPro-RCMI-1505 trial: multicenter study evaluating the use of a biodegradable balloon for the treatment of intermediate risk prostate cancer by intensity modulated radiotherapy; study protocol

**DOI:** 10.1186/s12885-018-4492-5

**Published:** 2018-05-16

**Authors:** David Pasquier, Emilie Bogart, François Bonodeau, Thomas Lacornerie, Eric Lartigau, Igor Latorzeff

**Affiliations:** 1 Academic Department of Radiation Oncology, Centre Oscar Lambret, Lille, France; 2 CRISTAL UMR CNRS 9189, Lille University, France; 3 Department of Biostatistics, Centre Oscar Lambret, Lille, France; 4 Department of Radiology, Centre O. Lambret, Lille, France; 5 Department of Medical Physics, Centre O. Lambret, Lille, France; 6 Department of Radiotherapy, Groupe ONCORAD Garonne, Clinique Pasteur, Toulouse, France

**Keywords:** Implantable biodegradable balloon, Rectal spacer, Intensity modulated radiotherapy, Prostate cancer

## Abstract

**Background:**

Prospective trials have demonstrated the advantage of dose-escalated radiotherapy for the biochemical and clinical control of intermediate risk prostate cancer. Dose escalation improves outcomes but increases risks of urinary and bowel toxicity. Recently the contribution of “spacers” positioned in the septum between the rectum and the prostate could improve the functional results of intensity modulated radiation therapy (IMRT). To date most of the evaluated devices were polyethylen glycol (PEG) and hyaluronic acid (HA). Men on the Spacer arm had decreased bowel toxicity and less decline in both urinary and bowel quality of life as compared to Control men in a randomized trial.

**Methods:**

This is an interventional, multi-center study to evaluate the use of biodegradable inflatable balloon for patients with intermediate risk prostate cancer treated by IMRT (74 to 80 Gy, 2 Gy/fraction) with daily image guided radiotherapy.

**Discussion:**

This multicenter prospective study will yield new data regarding dosimetric gain and implantation stages of Bioprotect balloon. Acute and late toxicities and quality of life will be registered too.

**Trial registration:**

NCT02478112, date of registration: 15/06/2015.

## Background

Prospective trials have demonstrated the advantage of dose-escalated radiotherapy for the biochemical and clinical control of prostate cancer. This benefit observed with three-dimensional conformational irradiation is counterbalanced by an increase of the urinary and digestive toxicities [1–4]. The Medical Research Council (MRC) conducted the MRC 01 randomized multicenter trial [2] comparing a conformal RT (2 Gy / session) delivering either 64 Gy or 74 Gy, in combination with neoadjuvant hormone therapy during 3 to 6 months. The 5-year biochemical relapse–free survival was 71% versus 60% (*p* = 0,0007) with 74 and 64 Gy respectively. In France the French Group for the Study of Uro-Genital Tumors (GETUG) conducted the GETUG 06 multicenter trial [3]. Dose escalation from 70 to 80 Gy provided a better 5-year biochemical outcome with slightly greater toxicity. Peeters and al. [4] reported the Dutch trial evaluating dose-response for 664 randomized patients in radiotherapy for prostate cancer. Patients were randomly assigned to a tridimensional conformal radiation treatment of either 68 Gy or 78 Gy (in 2 Gy fractions). The 5-year biological relapse–free survival was 54 and 64% respectively (*p* = 0.02). In these randomized trials dose escalation improved biological relapse free survival but was associated with higher rate of rectal toxicity.

There are no randomized trials comparing conformational three-dimensional conformational irradiation with intensity modulated radiation therapy (IMRT), but experiments conducted by several teams, including historically that of the Memorial Sloan-Kettering Cancer Center (MSKCC) [5] showed that it was possible to deliver increased radiation doses to the prostate while decreasing frequency of urinary and digestive complications of this “high dose” RT. However this approach imposes a very strict control of the position of the target volume (prostate) under the accelerator in order to translate this dosimetric advantage into clinical benefit. Image Guided Radiotherapy (IGRT) guarantees this positioning accuracy. First clinical benefits of using IGRT in combination with IMRT were published in 2012 by the MSKCC team in a retrospective analysis of 180 “IGRT” patients (fiducial markers implanted in the prostate and daily kV imaging) treated with 86.4 Gy, whatever the initial risk group, between 2008 and 2009 compared with a cohort of patients treated without IGRT between 2006 and 2007 [6]. Patients in high risk group in this study showed a significant improvement in biochemical control (from 77 to 97%, *p* = 0.041). For all analyzed patients, IGRT would lead to a significant reduction in urinary late toxicity: grade ≥ 2 toxicity rate decreased from 20 to 10.4% at 3 years thanks to the IGRT [6]. Despite significant technical advances (IMRT, IGRT) rectum dose remains a limiting factor in dose escalation. Although the role of moderate doses has been recently shown, severe toxicity is strongly related to high doses. Patients with V70 below or above 26% had a risk of grade 2 rectal morbidity of 13 and 54%, respectively [7].

Thanks to innovative techniques, rectal side effects could be reduced by moving the prostate away from the rectal wall through an injection of a biodegradable substance that creates a space in anterior perirectal fat. To date most of the evaluated devices were polyethylen glycol (PEG) and hyaluronic acid (HA). In Mok et al. review, a total of 11 studies involving human prostate cancer patients were identified in 6 studies using implants in patients treated with external beam radiotherapy and 5 studies treating patients with brachytherapy (BT). Four studies used PEG spacers, 5 used HA spacers, 1 study used implanted biodegradable balloons, and 1 study used collagen implants [8]. Prostate rectum (PR) separation created by the different PR spacers varied between 7 and 20 mm and was largely dependent on implantation protocol. The increased PR separation was associated with improved dosimetric rectal profiles. Relative reduction of V70 Gy ranged from 46 to 61%; V40 and V60 Gy were decreased too, from 40 to 65%. The use of prophylactic antibiotic therapy is estimated to reduce the risk of infection to less than 5% [8].

Outcomes following PEG spacer implantation was assessed by a prospective multicenter randomized controlled trial [9]. Computed tomography (CT) and magnetic resonance imaging (MRI) scans for treatment planning were used for 222 patients with prostate cancer with clinical stage T1 or T2. They were randomized to receive spacer implantation or no implantation (control). Image guided IMRT (79.2 Gy in 1.8-Gy fractions) was used. In this trial, spacer implantation was rated as “easy” or “very easy” in 98.7% of the patients. The hydrogel placement success rate was 99%. Overall acute rectal adverse event rates were the same between groups, but fewer spacer patients presented with rectal pain (*p* = 0.02). A significant decrease in late (3 to 15 months) rectal toxicity in the spacer group was noted (*p* = 0.04), with a 2.0 and 7.0% late rectal toxicity incidence in the spacer and control arms, respectively. At 6, 12, and 15 months, a lower ratio of spacer patients presented with bowel quality of life (QOL) decrease. 11.6% of spacer patients and 21.4% of control patients experienced 10-point decrease at 15 months (*p* = 0.087). Furthermore, at 6 months, 8.8% of spacer patients and 22.2% of control patients had 10-point urinary decreases (*p* = 0.003). At 3-years patients on the spacer group had less bowel toxicity and less decrease in both urinary and bowel QOL in comparison to control patients. On the control arm, 41% of patients presented with a detectable decline in bowel QOL (5-points) by patient reported outcomes, and 21% had a more serious decline (10-points). These rates were both reduced by 70% on the spacer arm (14 and 5%, respectively) [10].

The use of HA spacers in hypofractionated RT regimens were evaluated by Chapet et al. This phase II study aims to assess the rates of late rectal toxicities of grade ≥ 2 after hypofractionated radiotherapy of prostate cancer of 62 Gy in 20 fractions of 3.1 Gy with an HA spacer. Thirty-six patients with low- to intermediate-risk prostate cancer according to the D’Amico classification are included in the present protocol. As part of this phase 2 study, the patients received a 10 cm^3^ transperineal HA injection. HA spacer significantly reduced rectal wall dose and could allow a dose escalation from 6.5 Gy to 8.5 Gy per fraction without increasing the dose to the rectum. A phase 2 study is under way to assess the rate of acute and late rectal toxicities when SBRT (5 × 7.5 Gy) is combined with an injection of HA [11]. Other trials are currently evaluating rectal spacer in patients treated by stereotactic radiotherapy [https://clinicaltrials.gov/ct2/show/NCT02353832, https://clinicaltrials.gov/ct2/show/NCT02911922].

A biodegradable balloon can also be used: Bioprotect has designed an adapted device for this implantation procedure. Animal studies have confirmed its efficacy and also its good tolerance [12]. ProSpace® system is a deflated balloon made of a biodegradable polymer which is inserted perineally after hydrodissection thanks to an introducer.

The implantation procedure is performed under general anesthesia through a small perineal incision [13, 14]. A multi-institutional phase II study has been carried out in 6 centers using IMRT or 3D conformal RT [13]. Twenty three patients were analyzable and balloon was biodegraded within 6 months. The space between the prostate and rectum created by balloon implantation was about 2 cm, rising from 0.22 ± 0.2 cm to 2.47 ± 0.47 cm. This gap lasted during all the RT. In this first study three patients experienced acute urinary retention which resolved quickly following bladder drainage. In Melchert et al. [14] the prostate rectal wall separation resulted in an average reduction of the rectal V70% by 55.3% (±16.8%), V80% by 64.0% (±17.7%), V90% by 72.0% (±17.1%) in 26 patients.

## Methods

This is an interventional, multi-center study to evaluate the use of the BioProtect Balloon Implant System for patients with a prostate cancer of intermediate risk treated by intensity modulated radiotherapy.

### Study objectives

The main objective is to assess the dosimetric gain from the contribution of the implantable BioProtect balloon on organs at risk.

Secondary objectives are: evaluation of implantation stages of the balloon and its technical feasibility, evaluation of acute and late toxicities, correlation between the delay (between the implantation of the balloon and the radiotherapy) and the complications due to the balloon implantation, benefit from the Bioprotect Balloon Implant System compared to usual treatments for acute proctitis and measurement of the quality of life.

### Main inclusion and exclusion criteria are specified in Table 1

#### Evaluation criteria

##### Dosimetric gain from the implantable BioProtect balloon on organs at risk (OAR)

A dosimetric computed tomographic (CT) scan is systematically performed before (CT1) and after (CT2) the implantation. Several dosimetric criteria will be evaluated including near maximum rectal dose, rectum volume receiving 90 to 50% of the prescribed dose at 74-80 Gy. A relative reduction of 50% of the rectal volume receiving at least 70 Gy is the primary endpoint. Bladder wall doses will also be reported at V50 and V60. Patients will be treated with IMRT and daily IGRT, 2 Gy/fraction, total dose 74-80 Gy.

**Table 1:** Inclusion and exclusion criteria

Main inclusion criteria All the following must be met at the time of screening. – Patient over 18 years old – With a localized adenocarcinoma of the prostate: ○ of intermediate risk of D’AMICO ○ and of stage MRI < T3 – Requiring a treatment with Intensity Modulated Radiotherapy – PSA (Prostate-Specific Antigen) levels ≤20 ng/mL before external beam radiotherapy – Prostate volume > 15 cm^3^ – Short hormone therapy possibly associated (4-6 months) – Patient without clinical signs of progressive disease – Performance status ECOG (Eastern Cooperative Oncology Group) ≤ 1 – Life expectancy ≥10 years – Informed consent signed – Affiliation to a social security system	
Main exclusion criteria – Incompatibility to the implantation of a Bioprotect balloon: ○ ongoing anticoagulant by vitamin K antagonist (VKA) or heparintherapy ○ patient with immunosuppression or with serious chronic diseases such as heart failure, cirrhosis, chronic kidney failure, colic or rectal digestive inflammatory disease ○ history of prostatitis or of repeated prostatic resections ○ history of recto-colic inflammatory disease or of lower gastrointestinal infection ○ untreated perineal wound – Prior treatment with hormone therapy (> 6 months) – History of another invasive cancer within 5 years prior to study entry (excepted a treated basal cell skin carcinoma) – History of pelvic radiotherapy – Severe hypertension non controlled by an adapted treatment (≥ 160 mmHg in systole and/or ≥ 90 mmHg in diastole) – Ongoing antineoplastic therapy – Person deprived of liberty or under tutorship – Inability to submit to the medical monitoring of the study for geographical, social or psychological reasons. – Conformal radiotherapy without intensity modulation	

##### Implantation stages of the balloon and technical feasibility

Balloon implantation, difficulties for implantation, implant procedure time (mn) and radiotherapy delivery will be evaluated.

##### Tolerance evaluation

Implant-related toxicities and urinary and rectal toxicities due to irradiation will be evaluated according to the NCI-CTCAE v4.0 scale. Acute toxicities will be defined as occurring within the first 6 months after radiotherapy, and late toxicity when occurring beyond that period (Table 2).

**Table 2:** Study calendar

	Within 60 days before the first day of radiation therapy	Within 30 days before the first day of radiation therapy	Within 3 weeks before the first day of radiation therapy		During radiotherapy	At the end of treatment	Monitoring period	Study exit
			Before balloon implantation	After balloon implantation				
Clinical exams								
History of the disease, main health history, previous treatments	X							
Complete clinical examination with assessment of the general condition (Performance Status)	X				X 1 / week	X	X	X
IPSS score	X			X	X At mid-treatment	X	X At 3, 6, 12 et 24 months	X
Laboratory tests								
PSA level	X						X At 3 months then every 6 months	X
Quality of life								
EORTC-QLQ-C30 Questionnaire		X		X	X At mid-treatment	X	X At 3, 6, 12 et 24 months	X
EORTC-QLQ-PR25 Questionnaire		X		X	X At mid-treatment	X	X At 3, 6, 12 et 24 months	X
Paraclinical exams								
Tumor evaluation by computed tomography scanner			X					
Full description and measurements according to RECIST			X					
Dosimetric computed tomography scanner			X	X				
Treatments								
Side effects related to radiotherapy					X 1 / week	X	X At 3, 6, 12 et 24 months	X

The evaluation of the correlation between the delay (between positioning of the device and radiotherapy treatment) and the occurrence of complications due to the device implantation will be done by using a Wilcoxon or Student test (depending on the nature of the variables).

##### Quality of life

Patients will complete quality of life questionnaires prior to the start of treatment, at mid-treatment, at the end of the radiotherapy and at 3, 6, 12 and 24 months after the end of the radiotherapy (Table 2).

QLQ-C30 and QLQ-PR25 questionnaires will be completed by the patient. The first questionnaire (before implantation) will be completed at the first consultation. During the first consultation, the patient will be asked to complete these on-line questionnaires on the secured platform AQUILAB Share Place. He will receive personalized access codes to connect on line and fill out these questionnaires (Table 2). The results will be accessible to the physician on the platform.

##### Evaluation of urinary symptoms

The IPSS (International Prostate Score Symptom) was designed to be self-administered by the patient. Its French version has been validated. It is based on the answers to seven questions concerning urinary symptoms. The total score can therefore range from 0 to 35 (asymptomatic to very symptomatic). Symptoms are categorized as follows: mild (symptom score less than or equal to 7), moderate (symptom score range 8-19) or severe (symptom score range 20-35).

### Statistics

#### Number of patients

In literature rectal spacers allows a relative reduction of about 50% of the rectal volume receiving at least 70Gy. The sample size of 50 (in order to obtain 44 evaluable patients) was calculated to have a 90% power at 5% statistical significance level to detect an irradiated volume difference V70 of 5 (decrease from 10 to 5%) with a standard deviation of 10. V70 with and without the balloon implantation will be compared using equality test on matched data: student test if data are normal or Wilcoxon test if data are non-normal.

### Description of the device

#### BioProtect balloon implant

The BioProtect biodegradable Balloon Implant consists of a biodegradable inflatable balloon (mounted on a deployer) and an installation kit (echogenic needles, dilator and introducer sheath). It is provided as a sterile device. Once the balloon is in situ, it is inflated with sterile saline thanks to a plastic Luer-Lok syringe (20 ml or 50 ml). Users of this system must have been trained and certified by a Bioprotetc’s physician trainer before using the balloon. The implantation is performed under general anesthesia or neuroleptanalgesia. In case of difficulty during penetration of the perineum skin, a small incision (3-5 mm) using a scalpel at the point of insertion can be made. A minimum delay of 7 days is required between the implantation and the second computed tomography scanner.

#### Patient preparation

Two days before the implantation and during 5 consecutive days, the patient must be administered a broad-spectrum antibiotic (oral fluroquinolone). Prior to the implantation, patients must also use a bowel preparation (laxative).

It is recommended to introduce a urethral catheter into the bladder at the beginning of the session to empty the bladder and to help balloon positioning.

## Discussion

Literature suggests that rectal spacer may reduce rectal side effects after prostate cancer radiotherapy. Several reports show important decrease of rectal dose [8]. Increased perirectal space using HA spacer reduced rectal irradiation, rectal toxicity severity, and decreased rates of patients experiencing declines in bowel quality of life in a randomized trial. Most of the series evaluated HA and PEG spacers. The aim of this study is to evaluate dosimetric gain, implantation procedure, acute and late toxicities with biodegradable BioProtect Balloon. The procedure seems a little more invasive than HA and PEG spacers, with the necessity of a short perineal incision. On the other hand, an advantage could be a better stability of this device, due to its inflatable balloon concept. The envelope prevents lateral and cranio-caudal dispersion of the product as observed sometimes with others spacers and could allow a more reproducible implantation.

## Abbreviations

BT: Brachytherapy
CT: Computed tomography
ECOG: Eastern Cooperative Oncology Group
GETUG: French Group for the Study of Uro-Genital Tumors
HA: Hyaluronic acid
IGRT: Image guided radiotherapy
IMRT: Intensity modulated radiation therapy
IPSS: International Prostate Score Symptom
MRC: Medical Research Council
MRI: Magnetic resonance imaging
MSKCC: Memorial Sloan-Kettering Cancer Center
PEG: Polyethylen glycol
PR: Prostate rectum
PSA: Prostate-specific antigen
RTP: Radiation treatment planning
VKA: Vitamin K antagonist
